# Imaging gait analysis: An fMRI dual task study

**DOI:** 10.1002/brb3.724

**Published:** 2017-07-21

**Authors:** Céline N. Bürki, Stephanie A. Bridenbaugh, Julia Reinhardt, Christoph Stippich, Reto W. Kressig, Maria Blatow

**Affiliations:** ^1^ Division of Diagnostic and Interventional Neuroradiology Department of Radiology University of Basel Hospital and University of Basel Basel Switzerland; ^2^ Felix Platter‐Hospital University Center for Medicine of Aging and University of Basel Basel Switzerland

**Keywords:** cognitive aging, cognitive‐motor dual tasking, fall risk, geriatric clinical diagnostics, superior parietal lobe

## Abstract

**Introduction:**

In geriatric clinical diagnostics, gait analysis with cognitive‐motor dual tasking is used to predict fall risk and cognitive decline. To date, the neural correlates of cognitive‐motor dual tasking processes are not fully understood. To investigate these underlying neural mechanisms, we designed an fMRI paradigm to reproduce the gait analysis.

**Methods:**

We tested the fMRI paradigm’s feasibility in a substudy with fifteen young adults and assessed 31 healthy older adults in the main study. First, gait speed and variability were quantified using the GAITRite^©^ electronic walkway. Then, participants lying in the MRI‐scanner were stepping on pedals of an MRI‐compatible stepping device used to imitate gait during functional imaging. In each session, participants performed cognitive and motor single tasks as well as cognitive‐motor dual tasks.

**Results:**

Behavioral results showed that the parameters of both gait analyses, GAITRite^©^ and fMRI, were significantly positively correlated. FMRI results revealed significantly reduced brain activation during dual task compared to single task conditions. Functional ROI analysis showed that activation in the superior parietal lobe (SPL) decreased less from single to dual task condition than activation in primary motor cortex and in supplementary motor areas. Moreover, SPL activation was increased during dual tasks in subjects exhibiting lower stepping speed and lower executive control.

**Conclusion:**

We were able to simulate walking during functional imaging with valid results that reproduce those from the GAITRite^©^ gait analysis. On the neural level, SPL seems to play a crucial role in cognitive‐motor dual tasking and to be linked to divided attention processes, particularly when motor activity is involved.

## 1 INTRODUCTION

Walking is an implicit motor task which requires few attentional resources. However, gait alterations such as decreased walking speed and increased gait variability are associated with deficits in attentional processes (Baetens et al., 2012; Bridenbaugh & Kressig, 2011; Hausdorff, Balash, & Giladi, 2003). These gait alterations become especially prominent during a dual task condition when performing a concurrent cognitive task. Cognitive‐motor dual tasking can be highly demanding, especially for older adults. In their seminal study, Lundin‐Olsson, Nyberg, and Gustafson (1997) found that some older adults stopped walking in order to answer a simple question. The authors showed that 80% of these older adults fell at least once in the following six months, while older adults who continued walking while talking fell much less often. This documents the strong connection between motor performance, cognition and fall risk. Gait performance assessed during dual task conditions represents a marker for fall risk as well as cognitive disorders such as dementia (Amboni, Barone, & Hausdorff, 2013; Kressig, Herrmann, Grandjean, Michel, & Beauchet, 2008; Montero‐Odasso, Verghese, Beauchet, & Hausdorff, 2012; Verghese et al., 2014).

In recent years, electronic gait analysis has been used increasingly as a diagnostic tool for the evaluation of fall risk and cognitive impairment (Kressig & Beauchet, 2004). This assessment is mostly used in older patients potentially suffering from a form of dementia. Gait analysis assesses the degree to which gait is no longer an automatic and purely procedural motor task and, therefore, requires additional attentional control. Importantly, gait analysis with walking as a single‐task condition alone is often insufficient to reveal deficits. The use of a dual‐task paradigm, walking while concurrently performing a cognitive task, is required to assess the effects of divided attention on gait control and cognitive performance (Allali et al., 2007). When, for instance, a healthy older adult is asked to walk while simultaneously naming animals, walking speed is generally decreased and step‐to‐step variability is increased compared to walking without performing an additional task (Springer et al., 2006). The most sensitive marker of gait analysis is the difference in certain gait parameters between the single normal walking task (i.e., habitual, self‐selected speed) and a cognitive‐motor dual task (normal walking and a simultaneously performed cognitive task), the so called dual task costs or cognitive‐motor interference. Larger dual‐task costs represent greater severity of impairment. Larger changes in gait parameters than those commonly observed in healthy older adults are associated with mild cognitive impairment, Alzheimer's disease, Parkinson, or with an increased fall risk (Bridenbaugh & Kressig, 2015). Cognitive‐motor interference assessed with the cognitive‐motor dual task paradigm is also very sensitive for detecting decrements in neurochemistry and volume of the primary motor cortex and provides diagnostic information about mobility decline and falls (Annweiler et al., 2013).

To date, little research is available on the neural correlates of real or imitated gait using high spatial resolution imaging methods such as functional magnetic resonance imaging (fMRI). There are some fMRI studies in poststroke patients which investigated lower limb movements (Dobkin, Firestine, West, Saremi, & Woods, 2004; Enzinger et al., 2008; Luft et al., 2005; Promjunyakul, Schmit, & Schindler‐Ivens, 2015). These paradigms were not developed to imitate gait, rather to compare right and left leg movements sequentially. However, they all assessed lower limb movement, which is involved in imitated gait paradigms. The studies report mostly consistent areas of brain activation for lower limb movements, namely: primary and secondary motor and sensory cortices, supplementary motor area, cingulate motor area, cerebellum and basal ganglia. These studies reported on real or imitated gait as a single task.

Evidence on neural activation during real or imagined gait and cognitive‐motor dual tasking is limited and inconsistent. In their recent review on brain activation during walking and cognitive‐motor dual tasking, Hamacher, Herold, Wiegel, Hamacher, and Schega (2015) reviewed a wide range of studies which included the imaging methods functional near‐infrared spectroscopy (fNRIS), electroencephalography and positron emission tomography during real walking and fMRI during imagined walking. They identified a large number of involved brain regions and were able to classify them into either a direct or an indirect locomotion pathway. The direct locomotion pathway allows locomotion via primary motor cortex, cerebellum and spinal cord while the indirect pathway regulates locomotion via prefrontal cortex, premotor areas and the basal ganglia. In particular, more complex, goal‐directed and dual task motor activations are associated with the indirect pathway as well as increased activation in the fronto‐parietal network including cingulate cortex, parietal areas and insula. The seven studies Hamacher et al. (2015) reviewed specifically on cognitive‐motor dual tasking revealed differences in brain activation patterns between single and dual tasks. The findings were contradictory and only two studies regarding brain activation in healthy older adults were available: One study reported increased prefrontal activation during dual tasking (Holtzer et al., 2011) while the other reported decreased prefrontal activation (Beurskens, Helmich, Rein, & Bock, 2014).

It is well‐known that the execution of cognitive tasks is associated with brain activation in a typical neural cognitive control network. This network consists of a set of coactive fronto‐parietal cortical regions (Cole & Schneider, 2007; Dosenbach et al., 2007; Niendam et al., 2012) that is, anterior cingulate cortex/presupplementary motor area, dorsolateral prefrontal cortex, inferior frontal junction, anterior insular cortex, dorsal premotor cortex, and posterior parietal cortex. Several studies found evidence that this network is also involved in cognitive‐motor dual task paradigms (Rémy, Wenderoth, Lipkens, & Swinnen, 2010; Wu, Liu, Hallett, Zheng, & Chan, 2013).

In summary, literature on brain activation during cognitive‐motor dual tasking is sparse, particularly in older adults. There is some evidence that in cognitive‐motor dual tasks the fronto‐parietal cognitive control network is involved. However, there is no consensus about whether brain activation increases or decreases during dual tasking compared to single tasking. Moreover, direct comparison of study results of the literature available on brain activation during cognitive‐motor dual tasking is difficult because of the different tasks investigated.

Nijboer, Borst, van Rijn, and Taatgen (2014) argued that no general activation difference pattern exists between single and dual tasks, rather the nature of the two concurrently performed tasks is crucial for the resulting brain activation patterns. The authors proposed a dual‐task interference and time‐sharing hypothesis: All dual‐task situations require control of interference and switching between two competing tasks. The amount of resource overlap between the two tasks determines the neural activation pattern. The more the resources or processes overlap, the larger is the interference between the two tasks. Large dual task interference entails a large overlap of activated brain regions. Nijboer et al. (2014) proposed that the greater the dual‐task interference, the more the amount of brain activation in the overlapping brain regions cumulate during a dual task condition. Nonadditive activations are primarily seen in areas used by just one of the tasks. According to the time‐sharing hypothesis, all available time has to be shared between tasks: resources required by just one task can thus be accessed less frequently, leading to decreased activation during dual tasking.

The purpose of this study was to advance our understanding of gait analysis in older adults by imaging areas of brain activation using fMRI during cognitive‐motor dual tasking. We were particularly interested in imaging the brain activation in older adults because little is known about the neuro‐motor control of gait and the association of gait changes and cognitive decline in this population. Older age is normally accompanied by cognitive decline, which is observed in multiple cognitive domains such as memory and attention (e.g., Lövdén, Ghisletta, & Lindenberger, 2004). A better understanding of the neuro‐motor control of gait in older adults could contribute to the development of clinical tools for the early diagnosis of cognitive decline or dementia.

The first crucial question was how brain activation changes from single to dual task. The second question was whether there are one or more brain regions that are sensitive to deficits in cognitive‐motor dual tasking and may serve as target regions of interest for future research and diagnostics in older adults. In contrast to group analysis‐based studies, we aimed to identify the target regions at the individual level and, therefore, applied an individual fMRI analysis routine also used for diagnostic purposes (Blatow et al., 2011; Stippich, Blatow, & Krakow, 2007).

To investigate the neural correlates of gait analysis we developed an fMRI paradigm to simulate gait analysis as accurately as possible. In a substudy we tested the feasibility of the fMRI paradigm in younger adults. In the main study, we included a sample of older adults and tested the fMRI paradigm’s validity. First, we compared the behavioral data of the fMRI paradigm and the gait analysis. Second, we investigated the neural correlates of cognitive‐motor dual tasking and extracted a target region of interest as a potential neuronal marker for deficits in cognitive‐motor dual tasking in older adults. Third, we analyzed the relationship between the individual brain activation and parameters from gait analysis and other cognitive tests.

## 2 METHODS

### 2.1 Participants

Fifteen younger volunteers participated in the substudy in which only the second experimental session, i.e., the fMRI paradigm, was conducted (see section Procedure; mean age ± SD: 27.9 ± 4.44, 9 females). Thirty‐one older volunteers aged 70 or older took part in the main study (mean age ± SD: 75.83 ± 4.27, 14 females). Participants had no history of neurological or psychiatric disorders and reported themselves as healthy. All participants were right‐handed according to the test of handedness from Annett (1967). All subjects gave written informed consent prior to the experimental sessions. The study was approved by the local Ethical Committee Basel, Switzerland.

### 2.2 Materials

#### 2.2.1 Behavioral tasks

**Figure 1:**
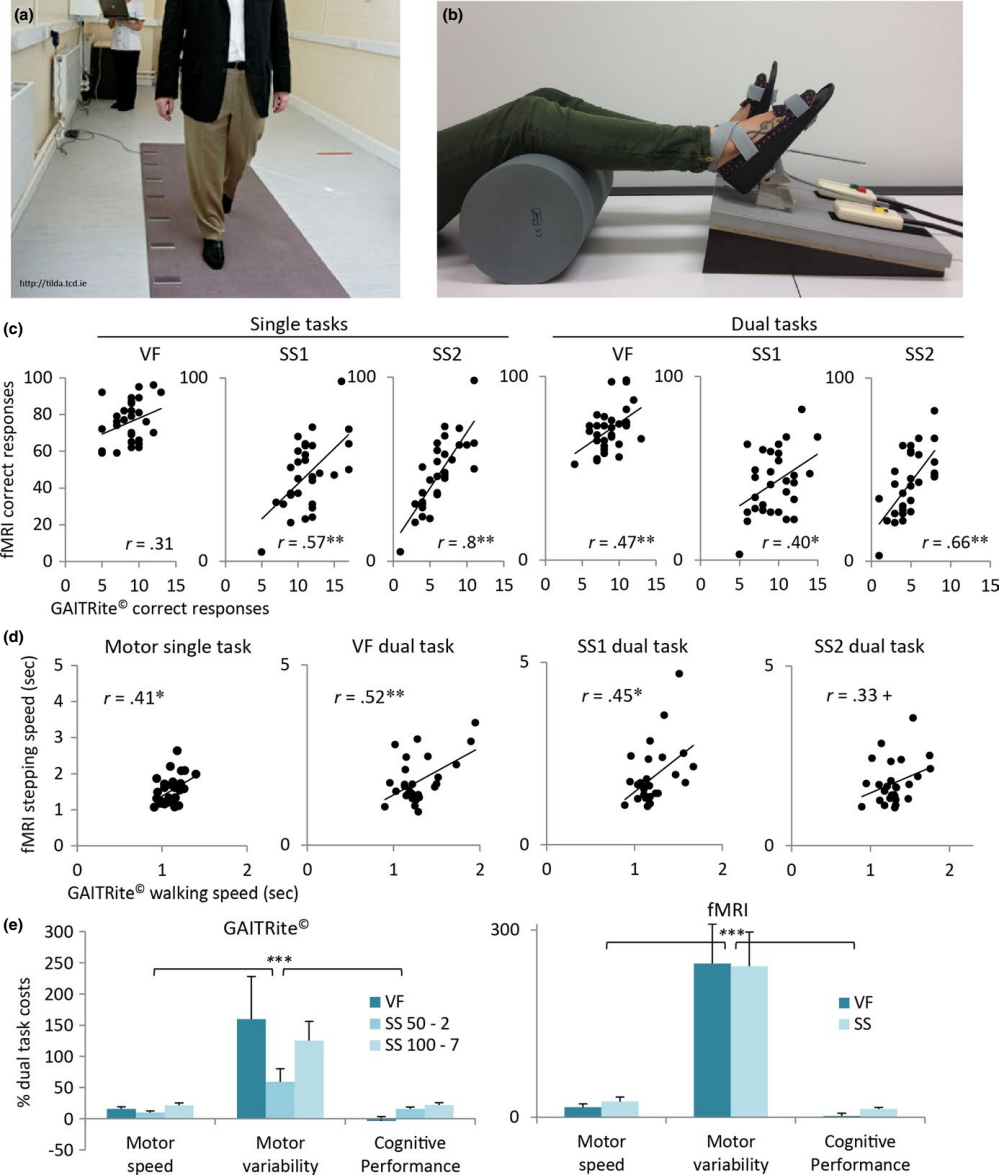
(a) GAITRite^©^ electronic walkway system with embedded pressure sensors which register the walking parameters. (b) MRI‐compatible stepping device, stepping parameters are recorded by response pads. (c) Positive and mainly significant correlations between the total number of correct responses of the GAITRite^©^ and the fMRI gait analysis. (d) Positive and significant correlations between walking or stepping speed in sec of the GAITRite^©^ and the fMRI gait analysis. (e) Mean and SE percent dual task costs. The dual task costs were significantly larger for motor variability than for motor speed and cognitive performance; this was true for both gait analyses. VF = verbal fluency; SS = serial subtraction; ***p <* *.001; **p <* *.01; *p <* *.05; + *p *<* *.1

The gait analysis was employed using the GAITRite^©^ electronic walkway system of approximately ten meters in length (Figure 1a). The walkway is equipped with embedded pressure sensors which register spatial and temporal parameters of the entire gait pattern. The gait analysis procedure included the assessment of, firstly, walking at normal (habitual, self‐selected) pace (motor single task). Subsequently, three cognitive‐motor dual tasks were employed: walking at normal pace while naming animals (verbal fluency dual task), while counting backwards out loud from 50 by twos (serial subtraction dual task 1) and while counting backwards out loud from 100 by sevens (serial subtraction dual task 2). Finally, the performance in the cognitive single tasks while sitting was assessed (naming animals, counting backwards), using the time each participant needed for the corresponding dual task. We used the following dependent variables for further analyses: number of correct responses corrected for walking time (cognitive performance), mean cycle time (also known as stride time) walking speed in seconds (motor speed) and walking cycle time variability calculated as coefficient of variation (SD/M cycle time × 100). For the SD and means in calculations for the cycle time variability, values from both the right and the left legs were used ((mean of right leg cycle time variability + mean of left leg variability)/2). Additionally, we calculated the dual task costs as the difference between single and dual task performance relative to the single task performance ((single task − dual task)/single task × 100) in order to estimate reserve capacity. To investigate the relationship of the gait analysis and cognitive‐motor dual task brain activation with performances in other cognitive domains, several neuropsychological tests were employed after the GAITRite^©^ gait analysis; a list of the tasks and references is provided in Table 1. To test whether gait analysis performance is associated with executive functions and interference management we conducted the Trail Making Test A and B (TMT) and the Stroop task. For the assessment of working memory performance we included verbal and visuo‐spatial span tasks and a 2‐back task, for processing speed a simple reaction time task and for general fluid intelligence performance we conducted Raven's Progressive Matrices. All mean values of the neuropsychological tests were age‐appropriate.

**Table 1:** Neuropsychological tests conducted with the sample of older adults

Task	Cognitive performance	Dependent variable	Mean (SD)	References
Trail making test (TMT) Form A & B	Executive functions: Task switching	Percent task switching costs for task completion times: (B‐A)/A*100	166 (111)	Reitan (1966)
Stroop test	Interference, inhibition	Percent interference costs for task completion times: (incongruent‐color)/color *100	96 (41)	Spreen & Strauss (1998)
Verbal span (VeS) forwards VeS backwards Visual span (ViS) forwards ViS backwards	Verbal and spatial short term memory and working memory	Total number of correctly recalled items	6.94 (1.73) 6.42 (1.52) 7.65 (1.66) 7.48 (1.79)	Härting et al. (2000)
Verbal 2‐back task	Verbal working memory	Mean reaction time for targets in msec	856 (198)	Braver et al. (1997), Chicherio (2006), Ludwig et al. (2008), Owen et al. (1999)
Simple reaction time task	Processing speed and variability	Mean reaction time in msec and coefficient of variation (SD/M)	345 (48) 0.25 (0.11)	de Ribaupierre, Fagot, & Lecerf (2011)
Raven's progressive matrices	Fluid intelligence	Total number of correct responses	37.61 (7.08)	Raven (1958)
Montreal cognitive assessment (MoCA)	Cognitive screening	Total number of correct responses	27.61 (1.67)	Nasreddine et al. (2005)

#### 2.2.2 fMRI task

For the fMRI gait analysis paradigm, we developed an MRI‐compatible pedal which allows controlled foot movements and registers these during scanning (Figure 1b). A similar approach for registering foot movements during fMRI was proposed by Shine, Ward, Naismith, Pearson, and Lewis (2011) and Shine et al. (2013). A special fixture attached to the pedals sent pedal stepping times to the MRI‐compatible response pads (Lumina, Cedrus, USA). Stepping times were registered using the software Presentation (Version Neurobehavioral Systems, Inc., USA; RRID:SCR_002521). The stimuli (described below) were projected on a screen behind the scanner which the participants were able to see in a mirror attached to the head coil. We used a block design which comprised five different runs; each run was composed of five blocks of 18 sec baseline measures and four blocks of 36 sec stimulation measures. The baseline blocks consisted of a black fixation cross on a white screen. Each run started and ended with a baseline block. Between baseline and stimulation the blocks were alternated. In the first run, participants had to step on the pedals at their self‐selected normal walking pace (motor single task). During the stimulation blocks they saw a symbol of feet on the screen prompting them to step. The symbol was stationary and in no way suggested a stepping cadence. In the second run, participants had to step on the pedals and simultaneously name as many words as possible from given categories (verbal fluency dual task; e.g., fruits, names, vehicles, clothing items). Within each block, three different categories were presented for 12 sec for each task. The answers were registered by an MRI‐compatible microphone (Fiber Optic Microphone for fMRI, Optoacoustics, Israel). The third run consisted of stepping on the pedals while counting backwards out loud by sixes or sevens (serial subtraction dual task, e.g., 124–7, 111–6). Within each block, three different computational tasks were presented for 12 sec for each task. Then, in runs four and five, the two cognitive tasks were conducted as a single task without stepping on the pedals (verbal fluency task and serial subtraction task). The stimuli of the verbal fluency and the serial subtraction tasks in the dual task and in the single task condition were presented in a random order within the tasks. The order of presentation of the verbal fluency task and the serial subtraction task was counterbalanced between participants. We used the following behavioral dependent variables for further analyses: number of correct responses (cognitive performance), mean cycle time stepping speed in seconds (motor speed) and stepping variability calculated as coefficient of variation as well as dual task costs for these dependent measures (for calculations see above).

### 2.3 Procedure

Prior to the testing sessions, the participants were screened by phone to determine handedness, neurological or psychiatric disorders and a MoCA score of 26 points or more. After screening, mean MoCA score was 27.61 points (see Table 1). In the first session, participants came to the Basel Mobility Center of the Felix Platter‐Hospital in order to perform the gait analysis and the neuropsychological tests as described above. In the second session, participants were received at the Department of Radiology of the University of Basel Hospital for the MRI session. The participants first practiced the fMRI paradigm outside the scanner in a separate room sitting on a chair. The practice was continued until the participant understood the entire paradigm. Then, the participants were positioned for lying in the scanner with the feet fixed on the pedals and a cylindrical cushion placed under the knees for comfort. Movement artifacts were minimized by fixing the head with preformed foam cushions and by instructing each volunteer to gaze at a fixation point.

We first explored the feasibility of the fMRI gait analysis in younger participants. The younger volunteers participated only in the MRI session and did not undergo a GAITRite^©^ gait analysis. Due to technical difficulties, valid behavioral fMRI data was collected only for part of the younger participants, the data is, therefore, not reported here. The older volunteers participated in both testing sessions.

### 2.4 fMRI data acquisition and preprocessing

High‐resolution T1‐weighted 3D MRI images of the brain (magnetization‐prepared rapid acquisition of gradient echo sequence: repetition time 1570 msec, echo time 2.67 msec, 1 mm^3^ isotropic resolution, flip angle 9°, 192 contiguous sagittal slices, matrix size 256 mm) were acquired at 3 Tesla (Magnetom Verio, Siemens, Erlangen, Germany) with a 12‐channel head coil. Additionally, block‐designed blood‐oxygen‐level‐dependent (BOLD) fMRI (echo planar imaging sequences, 38 oblique slices parallel to the AC‐PC plane, slice thickness 3 mm, gap 1 mm, repetition time 2570 msec, echo time 30 msec) were performed.

MRI images were analyzed using the Brain Voyager software (Version 2.8; Brain Innovation, Maastricht, The Netherlands; RRID:SCR_013057). Preprocessing of the data included motion correction, temporal smoothing and a voxel‐wise calculation of BOLD activation using linear cross‐correlations (General Linear Model [GLM]). Data processing was fully standardized except for the manual overlay of functional images on structural MRI images and for the individual definition of reference points required for spatial normalization. All individual datasets were transformed to Talairach space (Talairach & Tournoux, 1988).

### 2.5 Statistics

#### 2.5.1 Behavioral data

Behavioral data were analyzed using IBM SPSS Statistics 23 (RRID:SCR_002865). We correlated data from the GAITRite^©^ and the fMRI gait analysis to test the validity of our fMRI gait analysis. We defined values beyond three standard deviations from the mean as outliers and discarded them from the correlation analyses. Figure 1c,d shows that the correlation between the GAITRite^©^ and the fMRI gait analysis is positive and largely significant. In addition we found that dual task costs were comparable between both gait analyses (Figure 1e). The repeated measures analysis of variance (ANOVA) including the factors measure (mean motor speed, motor variability and cognitive performance) X task condition (verbal fluency, serial subtraction) revealed a significant effect of measure for both gait analyses. This implies that dual task costs were significantly larger for motor variability than for motor speed or cognitive performance (main effect measure for GAITRite^©^: *F*_2,29_ = 15.76, *p *<* *.001; main effect measure for fMRI: *F*_2,27_ = 17.05, *p *<* *.001). The ANOVA measure X task condition X gait analysis (GAITRite^©^, fMRI; excluding the measures from the GAITRite^©^ gait analysis serial subtraction 50‐2) also showed that there was no significant interaction with gait analysis, but still a single significant main effect measures (*F*_2,27_ = 25.8, *p *<* *.001). This confirms the comparable results from both gait analyses. Thus, the fMRI gait analysis validly imitates the GAITRite^©^ gait analysis at the cognitive as well as at the motor level.

#### 2.5.2 fMRI data

Data were analyzed with Brain Voyager software individually using a single subject GLM analysis. In order to correct for motion artifacts, the motion correction parameters were included as confound parameters in the GLM analysis. Employing a dynamic threshold technique (Blatow, Nennig, Durst, Sartor, & Stippich, 2007; Blatow et al., 2011), individual centers of gravity and *t* values for defined regions of interest (ROIs) were determined. Group activation maps were computed using separate subjects fixed effects analysis. A repeated measures ANOVA was conducted with the individual *t* values (Age Group [younger, older] × ROI [M1 feet/tongue, SMA/CMA, SPL] × Task [motor single task, cognitive single task, dual tasks]) using SPSS. We also calculated percent dual task costs for *t* values for each ROI and conducted a repeated measures ANOVA (Age Group [younger, older] × ROI [M1, SMA/CMA, SPL]  × Task [cognitive, motor]). Finally, we correlated behavioral with fMRI activation data in order to assess the direct link between neural activation and behavioral performance.

## 3 RESULTS

**Figure 2:**
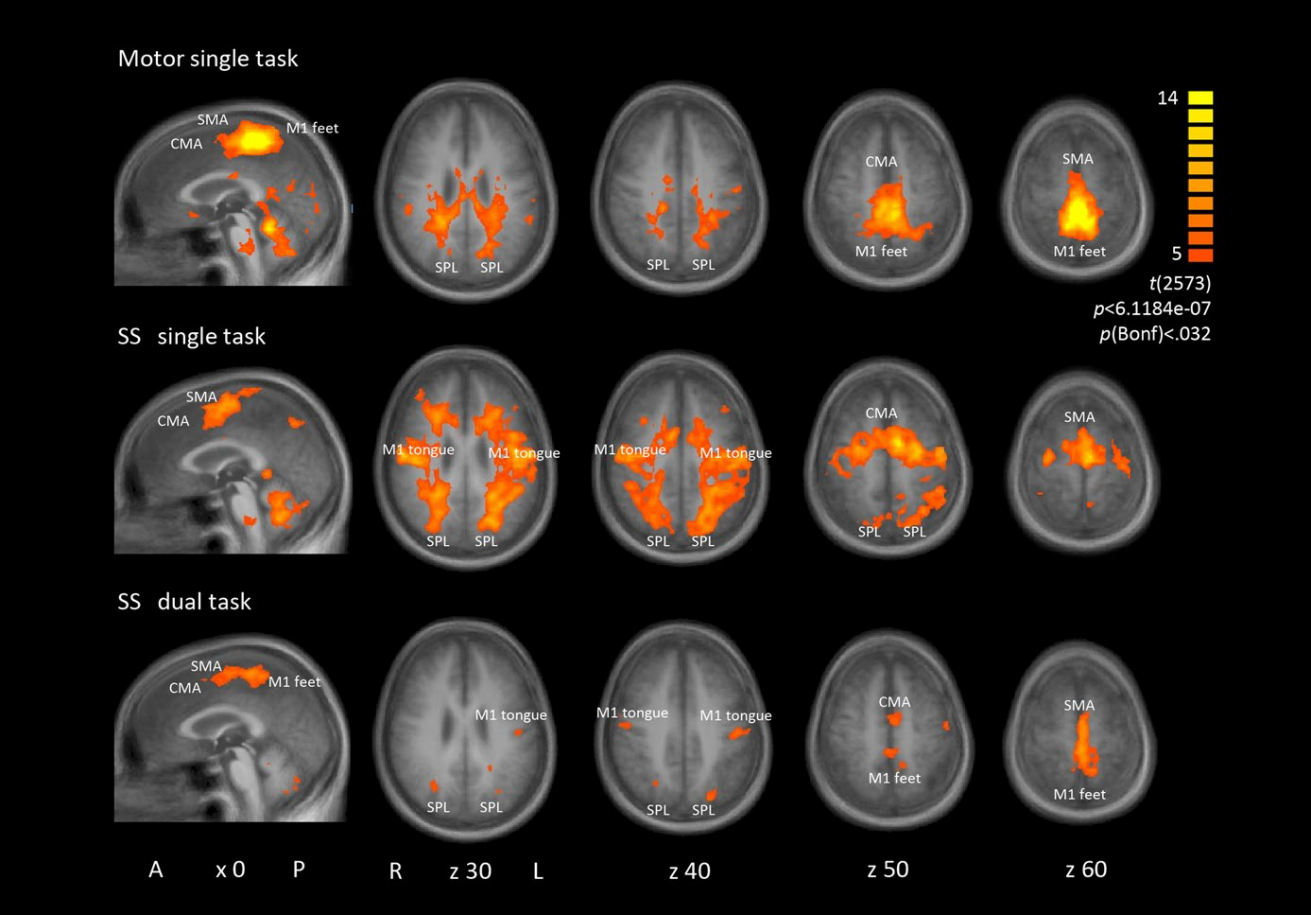
Group activation maps rendered onto sagittal and transversal group average brain slices (A = anterior, P = posterior, L = left, R = right; x, z = TAL coordinates). Group contrast *t* value maps of task versus baseline for motor single task, serial subtraction (SS) single task and SS dual task. Similar activation maps were found for the verbal fluency single and dual task. ROIs: primary motor cortex (M1), supplementary motor area (SMA), cingulate motor area (CMA), superior parietal lobe (SPL)

**Figure 3:**
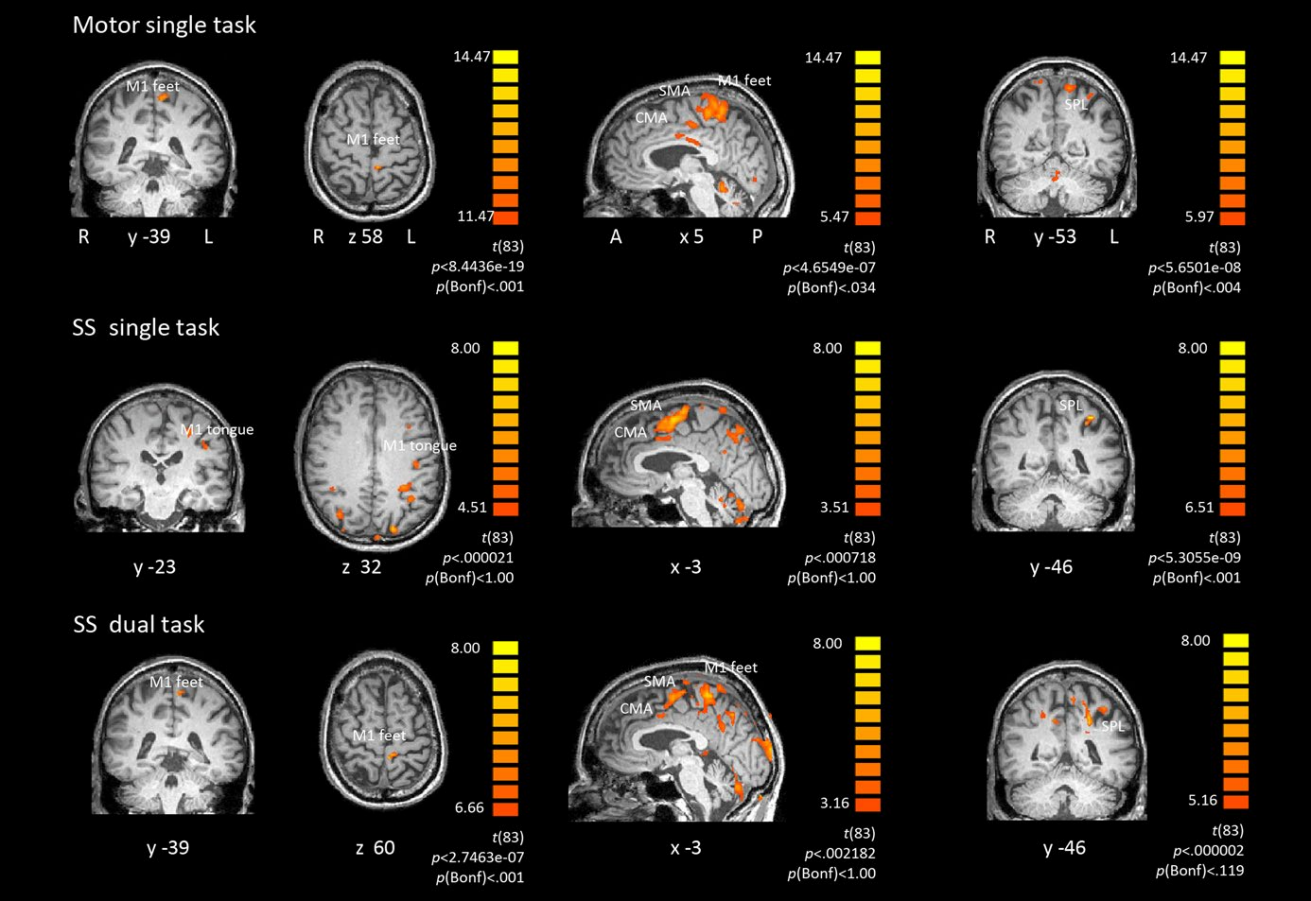
Individual activation maps rendered onto coronal, sagittal and transversal individual brain slices (L = left, R = right, A = anterior, P = posterior; x, y, z = TAL coordinates). Single subject contrast *t* value maps of task versus baseline for motor single task, serial subtraction (SS) single task and SS dual task. ROIs: primary motor cortex (M1), supplementary motor area (SMA), cingulate motor area (CMA), superior parietal lobe (SPL)

Figure 2 depicts brain activation at the group level for the different tasks. Using the same statistical threshold, more brain activation was found during single tasks than during dual tasks. The primary motor (M1) foot activation was not separable from supplementary motor area (SMA) and cingulate motor area (CMA) activation in the motor single task or in the dual tasks at group level. Similarly, SMA and CMA were not dissociable in all tasks at the group level and, in some cases, at the individual level (Figure 3).

ROIs were defined for all experiments; namely, the M1 areas of foot and tongue representations as well as SMA, CMA and superior parietal lobe (SPL) including intraparietal sulcus. All ROIs were defined in both brain hemispheres. The activations of SMA and CMA were often not distinguishable from each other or we found activation in one of the two ROIs. Since they are involved in the same motor‐associated processes we merged the SMA and CMA *t* values and named the new ROI SMA/CMA. We first analyzed the occurrence probability of activation within the ROIs in each task condition. Since only approximately 60% of the participants exhibited brain activation in the respective ROI during dual task conditions, we merged the values from both dual tasks and both single tasks in order to increase power. To do so, we used either the *t* value from one of the tasks or, whenever both values were available, we averaged the *t* values from both tasks.

In a second step, we investigated activation within each ROI at the individual level and extracted the individual *t* values. An example of an individual activation map using a dynamic threshold is shown in Figure 3.

**Figure 4:**
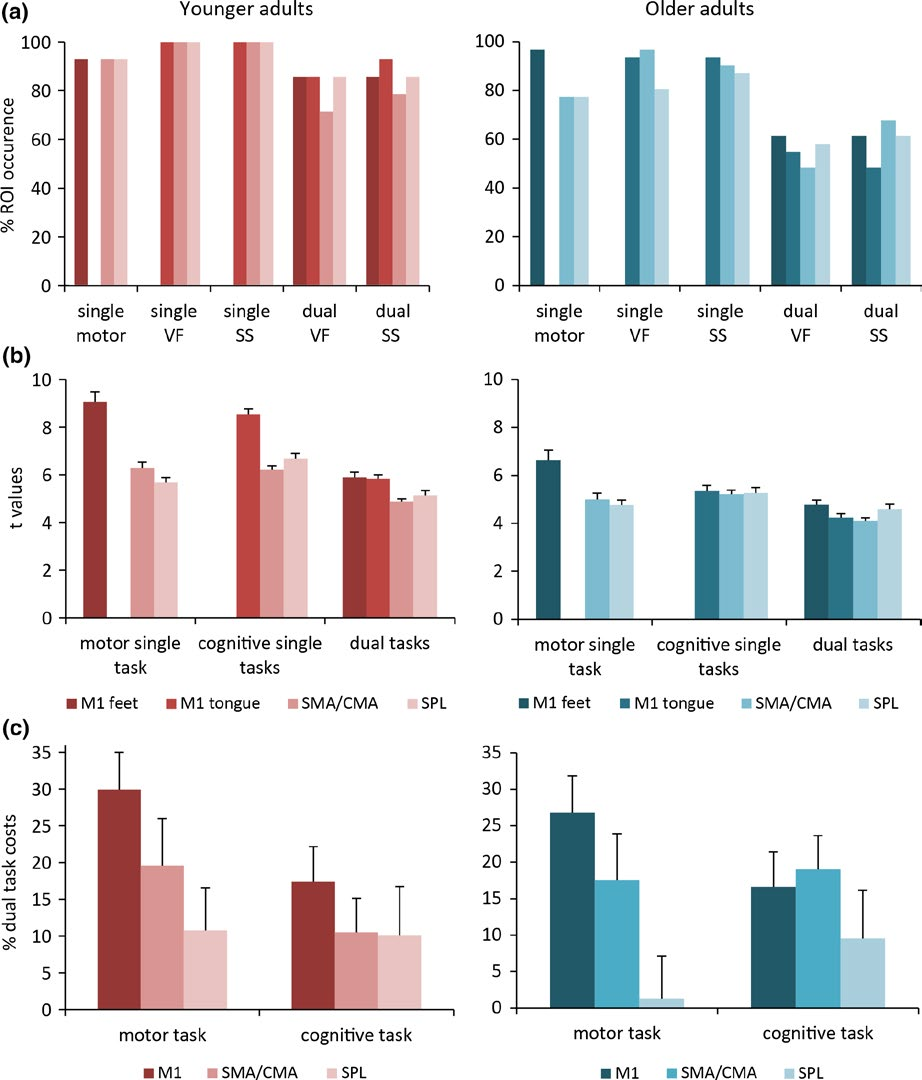
Data from younger adults in the left panels and from older adults in the right panels. (a) Percent occurrence of the ROI fMRI activation per task condition. ROIs: primary motor cortex (M1) for feet or tongue representation, supplementary motor area and cingulate motor area (SMA/CMA), superior parietal lobe (SPL). (b) Mean and SE *t* values of the fMRI activation per ROI of the contrast task versus baseline. The *t* values for the cognitive single tasks and the dual tasks are merged. (c) Mean and SE percent dual task costs: (single task – dual task)/single task × 100. The dual task costs are significantly lower for SPL than for the other ROIs. VF = verbal fluency; SS = serial subtraction

As Figure 4a shows, brain activation was not found for each ROI in each participant. In particular, older adults exhibited less brain activation than younger adults. It was more pronounced for the dual task conditions where only 50–70% of the older volunteers exhibited the respective activation. Regarding the *t* values (see Figure 4b), the ANOVA showed that activation was stronger in M1 than in the other ROIs (main effect ROI: *F*_2,40_ = 32.60, *p *<* *.001). Younger adults generally exhibited stronger brain activation than older adults (main effect age group: *F*_1,20_ = 9.70, *p *<* *.005). The analysis further revealed that the *t* values were larger during single task conditions than during dual task conditions (main effect task: *F*_2,40_ = 14.21, *p *<* *.001). This becomes evident in the dual task costs calculation which shows the percent activation change from a single task condition to a dual task condition (Figure 4c). The ANOVA showed a main effect of ROI (*F*_2,44_ = 9.15, *p *<* *.001), post hoc comparisons indicated that SPL exhibits significantly fewer dual task costs than M1 (*p *<* *.001), that is, less activation decrease from single to dual task. In the motor task, the dual task costs did not differ from zero in older adults. This indicates that SPL was activated to a similar amount in single and dual tasks and did not decrease in activation like the other ROIs. It seems, therefore, to play a special role in cognitive‐motor dual tasking, at least in older age. Overall no age differences in dual task costs were found.

**Figure 5:**
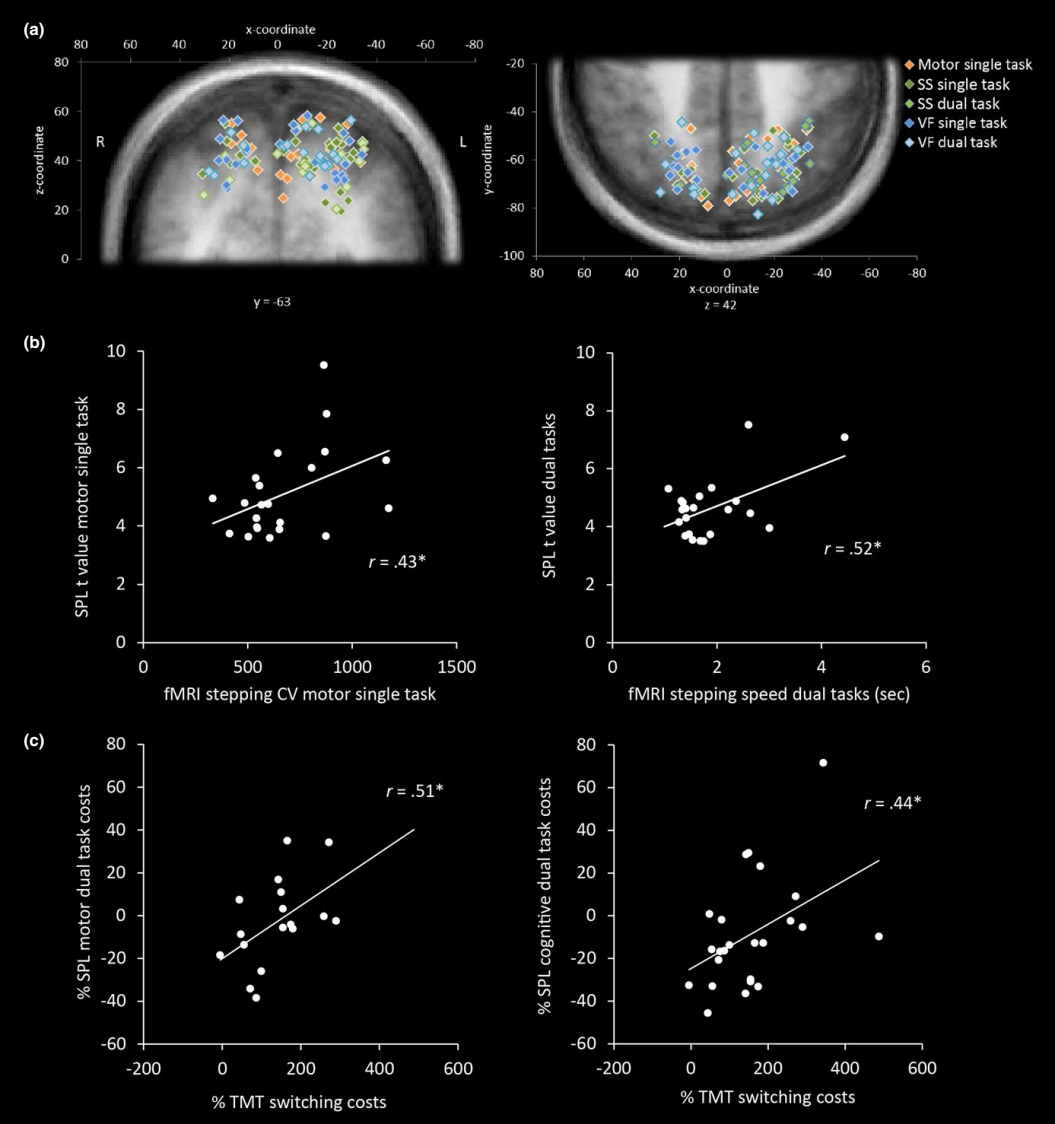
(a) Individual coordinates of the SPL ROI plotted on coronal (left) and transversal (right) group average brain slices. (b) Significant positive correlations between SPL *t* values and fMRI stepping coefficient of variation (CV) or mean cycle time for motor single task or merged dual tasks, respectively. (c) Significant positive correlations between percent dual task costs for SPL *t* values ((dual task – single task)/single task × 100) and percent switching costs in TMT ((B‐A)/A × 100). VF = verbal fluency; SS = serial subtraction; *p <* *.05

Figure 5a depicts the individual spatial coordinates of SPL activation for the different tasks. The spatial variability is large and SPL activation is distributed over the whole SPL and the intraparietal sulcus being predominant in the left hemisphere. We further investigated the association of SPL with behavioral measures in older adults. We correlated SPL values with fMRI stepping parameters and neuropsychological test performances. The analyses revealed significant positive correlations between stepping parameters of the fMRI gait analysis and SPL, indicating that the slower or more variable participants were stepping the larger was the SPL activation (Figure 5b). Furthermore, SPL dual task costs were also positively correlated with switching costs of the TMT, indicating that participants with large switching costs showed positive SPL dual task costs (Figure 5c). In other words, participants with large switching costs in TMT, and therefore low executive control performance, exhibited similar SPL activation in single and dual task or an increased activation in the dual task condition.

## 4 DISCUSSION

In this study we were able to reproduce the GAITRite^©^ gait analysis in an fMRI setting despite the challenging conditions of MRI. First, we had to develop a paradigm which can be performed in a lying position but is nevertheless as close as possible to actual walking. Second, we had to deal with movement artifacts which are produced by foot movements. Finally, we had to stimulate for a much longer duration (144 sec) than for the duration of a gait analysis (approx. 12 sec) on the electronic walkway in order to get a good fMRI signal. The results showed that high performers on the GAITRite^©^ walkway were also high performers in the scanner and vice versa. This was true for motor and for cognitive performance. Since we found the same behavioral dual task costs in both gait analyses, task prioritization seems to be independent of the test setting. Neither the cognitive nor the motor task was obviously prioritized on the gait walkway or in the scanner. The fMRI paradigm design had to take the potential difficulties elderly people may have in performing the tasks into account. Further, it needed to meet the requirements of a clinical fMRI protocol in order to be used later for patients with cognitive impairment (Blatow et al., 2011; Stippich et al., 2007). Therefore, runs were as short as possible, yet long enough to produce robust activation, task complexity was chosen to be potentially adaptable to individual subject's needs, and functional data was analyzed at the individual level.

Regarding brain activation, we found a generally decreased activation during dual tasks compared to single tasks. This result is in agreement with the dual‐task interference and the time‐sharing hypothesis which proposes that the amount of resource overlap between the two tasks determines the neural activation pattern (Nijboer et al., 2014). Since all available time has to be shared between the two tasks, resources required by just one task can be accessed less frequently. This leads to decreased activation during dual tasking. First, we found generally decreased brain activation in dual‐task conditions compared to single task conditions. The execution of walking and of performing a cognitive task requires different processes and therefore recruits different resources. The time has to be shared between the processes which results in an nonadditive brain pattern during dual tasking in our paradigm. Second, individuals exhibiting lower performance in executive function showed less nonadditive brain activation (in SPL) during dual tasking. This is also consistent with the time‐sharing hypothesis: the processes of lower performing individuals overlap more than those of higher performers; the activations are, therefore, more cumulated. Lower performing older adults experience more cognitive motor dual‐task interference than the higher performing ones as they rely more on cognitive resources for procedural memory tasks such as walking (Bürki, 2016; Lindenberger, Marsiske, & Baltes, 2000). This may be due to the so‐called cognitive permeation process which takes place in older age. The cognitive permeation hypothesis describes that, with advancing age, more cognitive resources have to be attributed to the compensation of sensory and sensorimotor deficits. In turn, fewer resources are available for intellectual tasks, as the already generally reduced resources in older adults have to be increasingly shared. So the resource overlap and competition between domains increases and compensatory resource allocation trade‐offs become more frequent (Li & Lindenberger, 2002). Therefore, sensory and sensorimotor processes, including procedural memory tasks such as walking or stepping, are no longer executed fully automatically but require increased cognitive resources and turn into more attentional control‐demanding tasks in older age. The progression of the cognitive permeation varies between older adults and leads to differing brain activation patterns between higher and lower performing older individuals.

Our findings agree with further studies, such as a recent EEG study, where counting backwards out loud while walking decreased frontal and parietal activity; whereas carrying a full glass of water while walking increased frontal activity in older participants (Marcar, Bridenbaugh, Kool, Niedermann, & Kressig, 2014). Walking and counting involve different resources which have to share the available time when performed simultaneously. Walking and carrying a glass of water involve several similar motor control resources which are accessed more frequently during dual task conditions. Our findings are also in accordance with results from a study by Just, Keller, and Cynkar (2008) which reports a decrease in brain activation during driving and performing a semantic task. The driving task and the semantic task do not show large resource overlaps. However, Rémy et al. (2010) report contrary results from cognitive‐motor dual tasking. They observed decreased activation mainly in overlapping brain regions during dual tasking. After some practice, however, the complex motor task was automatized and recruited other brain regions; the brain activation during dual tasking was no longer decreased.

Our findings revealed that brain activation was generally larger in younger adults than in older adults. This age‐related attenuation of BOLD signal has been reported in other age‐comparative studies on motor and cognitive brain activation (Hesselmann et al., 2001; Kannurpatti, Motes, Rypma, & Biswal, 2011). An opposite finding was that older adults exhibited larger brain activation than younger adults during task switching (Kunimi, Kiyama, & Nakai, 2016). However, age‐related BOLD signal differences have to be interpreted with care, since the vascular system may be different in older adults than in younger adults and, therefore, affect the BOLD response without necessarily reflecting a proportional change in the underlying neuronal signal (D'Esposito, 2006; D'Esposito, Zarahn, Aguirre, & Rypma, 1999).

Regarding the brain activation during dual tasking, we found the hypothesized fronto‐parietal activations of the cognitive control network (Cole & Schneider, 2007; Deprez et al., 2013). We did in particular expect increased prefrontal activation during dual tasking as observed by Holtzer et al. (2011). However, we were not able to find valid prefrontal activation in the majority of participants but only in few cases. This is most likely due to the short length of our fMRI paradigm, which was chosen to enable continuous stepping and, therefore, probably did not stimulate sufficiently to get a robust prefrontal BOLD signal.

Regarding the cognitive‐motor dual task costs at the brain level, SPL seems to be involved in a different way than the other ROIs. SPL activation is associated with multitasking and task switching performance and plays a crucial role when attention has to be divided among different processes (Al‐Hashimi, Zanto, & Gazzaley, 2015). It was further found to be related to motor imagery of gait and awareness and intention of movements during dual task condition (Bakker et al., 2008; Desmurget et al., 2009; Wagner, Shannon, Kahn, & Buckner, 2005). We identified the SPL as a core region of interest, which seems to be sensitive for individual differences in cognitive‐motor dual tasking. SPL activation was correlated with behavioral motor performance of the fMRI gait analysis. The more variable the participants were in the motor single task condition, the more the SPL region was activated. During dual task conditions, slowly stepping participants activated the SPL region to a higher degree. SPL seems to play an important role in motor coordination and control and has to be recruited to a higher degree when speed is decreased and variability is large, i.e., when stepping performance is lower. Furthermore, TMT switching costs were positively correlated with SPL dual task costs. This indicates that lower performing individuals did activate SPL to a larger, or at least similar, degree during dual tasks than during single tasks. This is a very interesting finding, since in all other ROIs the activation during dual tasking was decreased compared to the single tasks.

The TMT requires connecting test items according to a task switching rule by drawing lines between the items on a paper. The TMT is a measure of task switching performance and requires a motor response. It, therefore, also entails a motor component and can, in a broader sense, be defined as a cognitive‐motor dual task where a cognitive switching task is performed while the connection lines between test items have to be drawn manually as fast as possible. Switching costs are calculated between a nonswitching and a switching version of the task. TMT switching costs are associated with executive control performance (Sanchez‐Cubillo et al., 2009). Our findings revealed that those individuals exhibiting large TMT switching costs were those who showed similar or more SPL activation during a dual task condition as compared to a single task condition. This result enhances the explanation that low performing individuals, compared to high performing individuals, needed to activate SPL to a larger degree during dual tasking in order to compensate for lower cognitive control. This was true for both the motor and the cognitive tasks in the dual task paradigm. That leads to the hypothesis that SPL plays a bottle neck role in cognitive‐motor dual tasking.

An association between dual tasking and TMT performance has been reported before (Fritz & Basso, 2013; Mirelman et al., 2011; Siu, Chou, Mayr, Donkelaar, & Woollacott, 2008). Mirelman et al. (2011), for instance, reported better TMT performance in Parkinson's disease patients after dual task treadmill training. Furthermore, task switching requires cognitive control and has been found in many studies to rely on the cognitive control network (e.g., Jamadar, Thienel, & Karayanidis, 2015) which comprises SPL activation.

One limitation of this study is that the gait simulation in the MR scanner does not completely represent real gait, even though the paradigms correlate significantly. Due to the different gravitational load in these two conditions, proprioceptive and exteroceptive input to the brain is very different. This may limit the results for clinical application. However, until a vertical MRI scanner is developed which can scan the brain during standing or walking, we feel that the gait simulation presented in this manuscript comes one step closer to understanding brain function during gait.

## 5 CONCLUSION

This study shows the feasibility of dual task fMRI paradigms in older adults, yielding sufficiently robust activation to be analyzed at the individual level. This is a prerequisite for a potential use for diagnostic purposes, in particular in patients with cognitive impairment. In line with existing hypotheses, we found a general decrease in brain activation during dual tasks as compared to single tasks, reflecting network competition in processes of divided attention. We further identified SPL as a region sensitive to individual cognitive‐motor performance, making it a possible target region for future clinical research.

## CONFLICTS OF INTEREST

No potential conflicts of interest relevant to this article were reported.
